# Hypothesis: does adrenalitis caused by immune checkpoint-inhibitors put melanoma patients at an elevated risk for recurrence?

**DOI:** 10.1186/s40425-019-0651-8

**Published:** 2019-07-04

**Authors:** Igor Alexander Harsch

**Affiliations:** Department of Internal Medicine II, Division of Endocrinology and Metabolism, Thuringia Clinic Saalfeld “Georgius Agricola”, Rainweg 68, 07318 Saalfeld/Saale, Germany

**Keywords:** Immune checkpoint inhibitors, Malignant melanoma, Primary adrenal failure, Addison’s disease, ACTH, MSH, Dual-release hydrocortisone

## Abstract

Primary adrenal failure (Addison’s disease) is a rare complication of immune checkpoint inhibitor (ICI) therapy. Untreated – and also sometimes under adequate hydrocortisone replacement therapy – the levels of ACTH (Adrenocorticotropic hormone) and MSH (Melanocyte stimulating hormone) are elevated. This may be a reason for concern in patients with malignant melanoma (MM): Melanocortin receptors bind to ACTH and the different isoforms of MSH. For example, the melanocortin 1 receptor (MC1R) is overexpressed in many human melanoma cells. Since it is also involved in the proliferation of melanoma cells, the elevated levels of ACTH and its proteolytic cleavage product α-MSH typical for primary failure may lead to an activation of the receptor and, thus, put MM patients that suffered from primary adrenal failure after ICI therapy at an elevated risk for recurrence or an unfavorable course of the disease. Novel dual-release hydrocortisone therapy results in lower ACTH (and most probably lower α-MSH) levels due to the more physiological mode of hydrocortisone release. Given that the concern raised in this hypothesis is confirmed in future investigations, patients who suffer from primary adrenal failure after ICI therapy may benefit from a dual-release hydrocortisone replacement regimen.

Dear Editor,

From an endocrinologist’s point of view, I would like to share some interdisciplinary thoughts and concerns on primary adrenal failure (Addison’s disease) as a possible – yet rare – adverse event caused by immune checkpoint inhibitor (ICI) therapy [1]. These therapies are used increasingly to treat malignant melanoma (MM) and the results and benefits of the therapies are impressive. My point of concern is the possible side effect of an ICI-therapy mediated destruction of the adrenal gland in these patients:

It is well known that one typical feature of patients with Addison’s disease is the hyperpigmentation of the skin [2]. This is caused by the feedback mechanism after adrenal destruction causing the pituitary to produce more of the prohormone Proopiomelanocortin (POMC) which in turn dissociates into ACTH (Adrenocorticotropic hormone), γ-MSH (Melanocyte stimulating hormone) and β-Lipotropin. ACTH itself undergoes proteolytic cleavage to α-MSH. The plasmatic values of ACTH are typically increased in the early morning in patients and this can be the case even in the presence of an adequate hydrocortisone substitution therapy [3, 4]. Scott et al. [3] demonstrated such ACTH levels elevated from about 4–10 a.m.. These data were derived from a two-dose hydrocortisone replacement regimen (8.00 a.m. and 4.00 p.m.) in comparison to healthy volunteers. Compared to those, the most significant difference in the ACTH levels was observed between 8.00. and 9.00 a.m.. Even under a three-dose therapy regimen, due to the short half**-**life of hydrocortisone (approximately 1.5 h) with hydrocortisone taken in the morning at awakening, at lunch and in the afternoon [2] this will not change much, since an administration after 5 p.m. is not recommended to avoid sleep disturbance. In addition, the short-term feed-back induced by the steroid drugs determines large fluctuations of plasma ACTH levels [4]. These phenomena are explained by the observation that the replacement therapy cannot perfectly mimic the physiological rhythm of cortisol release. The elevated ACTH and MSH levels themselves are without pathophysiological effect in a patient with Addison’s disease. Their significance in patients with Addison’s disease and MM is unknown but may give reasons for concern:

All five melanocortin receptors that are known today can bind to ACTH and the different isoforms of MSH (α, β, γ) bind to different subtypes of these receptors. For example, the melanocortin 1 receptor (MC1R) binds to ACTH and all isoforms of MSH. This receptor is overexpressed on the cell surface of the majority of human melanomas [5]. MC1R is also involved in the proliferation of melanoma cells [6]. ACTH has a short half-live of about 6–7 min [7]. However, with the already mentioned elevated levels in the morning for some hours [3], these elevated levels may be of biological significance for this timespan, as well as the levels of its cleavage product α-MSH. The half-live of MSH varies with the degree of the acetylation of the N-terminal serine. The α-MSH is usually reported to have a plasma half-life of about 20 min in humans [8]. That said, it is at least theoretically conceivable that melanoma patients who suffer from Addison’s disease are at a higher risk of recurrence or unfavorable course of the disease. Fortunately, adrenalitis is still a rare side effect of the therapy with ICI [1] but may become more frequent with the increasing use of ICI therapies in MM that can be expected and the hypothesis may become testable then. Yet, the paucity of case reports do not allow conclusions concerning the issues addressed here [1].

In case of endocrine side effects, hypophysitis causing secondary adrenal failure is much more common. Patients who remain with an insufficiency of the adrenocorticotrophic axis after hypophysitis have no hyperpigmentation as those with primary adrenal failure since they have very low ACTH and α-MSH levels. Although not discussed under this hormonal aspect, it is highly interesting that a recent study suggested a positive correlation between hypophysitis and survival using ipilimumab for MM [9]. In their paper, the authors compared 17 patients with metastatic MM who had developed hypophysitis under ipilimumab therapy with 137 metastatic MM patients under the same therapy without hypophysitis. In a median-length follow-up of 11.5 months, hypopituitarism persisted in 13 of 17 patients with adrenal recovery in only one patient. The mean survival in the patient group that had had hypophysitis was 19.4 months, in the patients without hypophysitis it was 8.8 months. The authors discuss the incidence of hypophysitis as a positive predictor for survival. Of course, the case numbers preclude definite conclusions, but it is at least tempting to speculate about a protective role of the low ACTH levels in the patients with persistent secondary adrenal failure.

A clear distinction between the reasons for adrenal failure should be mandatory. A cortisone replacement therapy with a modified dual-release hydrocortisone is able to mimic the physiological conditions better. It has recently been demonstrated in M. Addison patients who were treated with 20 mg hydrocortisone thrice daily compared to a therapy with 20 mg dual-release cortisone that the decrease of ACTH levels was more marked and with a lower area under the curve in the latter group [10]. To my knowledge, there is no available data on the affinity and the binding of the melanocortin receptors for ACTH and the MSH subtypes in the context of coexisting MM and ICI-induced adrenalitis thus far. Until a reevaluation is available and possible, the therapy with dual-release cortisone, resulting in lower ACTH (and most probably lower α-MSH levels as its cleavage product, although not measured in this study) might be a wiser therapeutic approach in terms of hydrocortisone replacement therapy in MM patients with ICI-therapy induced primary adrenal failure.

## Data Availability

Not relevant, since hypothesis.

## Abbreviations

ACTH: Adrenocorticotropic hormone
ICI: Immune checkpoint inhibitor
MC1R: Melanocortin 1 receptor
MM: Malignant melanoma
MSH: Melanocyte stimulating hormone
POMC: Proopiomelanocortin
